# Refusing Medication Therapy in Involuntary Inpatient Treatment—A Multiperspective Qualitative Study

**DOI:** 10.3389/fpsyt.2019.00295

**Published:** 2019-05-09

**Authors:** Susanne Jaeger, Franziska Hüther, Tilman Steinert

**Affiliations:** ^1^Department of Psychiatry I, Ulm University, Centre for Psychiatry Suedwuerttemberg, Health Services Research Weissenau, Ravensburg, Germany; ^2^Department of Cardiovascular Surgery, Robert Bosch Hospital, Stuttgart, Germany

**Keywords:** compulsory treatment, involuntary treatment, coercion, medication refusal, qualitative analysis

## Abstract

**Objective:** Between June 2012 and February 2013, two decisions by the German Federal Constitutional Court restricted the so-far common practice to use involuntary medication in inpatients who were involuntarily hospitalized. Up to then, involuntary medication was justified by a judge’s decision on involuntary hospitalization. It could be applied according to clinical judgment even against the declared will of a patient. Since then, all domestic laws related to involuntary treatment had to be revised. For several months, involuntary medication was allowed only in an emergency. We were interested in the impact of the changed legal framework on the experiences of inpatients, their relatives, and clinical professionals during that time.

**Methods:** Thirty-two interviews were analyzed qualitatively using a grounded theory methodology framework.

**Results:** As a consequence of the restrictions to involuntary medication, special efforts by nursing and medical staff were required concerning de-escalation, ward management, and the promotion of treatment commitment in inpatients who refused medication. Family caregivers were also under strong pressure. They wanted to help and to protect their relatives, but some also welcomed the use of coercion if the patient refused treatment. Most of the interviewed patients had not even noticed that their rights to refuse medication had been strengthened. They complained primarily about the involuntary hospital stay and the associated limitations of their everyday lives. While patients and family members evaluated the refusal of medication from a biographical perspective, the mental health care professionals’ focus was on the patients’ symptoms, and they understood the situation from a professional perspective. It was obvious that, in any of the four perspectives, the problem of feeling restricted was crucial and that all groups strived to gain back their scope of action.

**Conclusion:** The temporary ban on involuntary medication questioned the hitherto common routines in inpatient treatment, in particular when patients refused to take medication. Each of the different groups did not feel good about the situation, for different reasons, however. As a consequence, it might be indispensable to increase awareness of the different perspectives and to focus the efforts on the establishment of nonviolent treatment structures and practices.

## Introduction

In 2011, the German Federal Constitutional Court imposed sharp restrictions on the use of compulsory treatment in mental health hospitals by two decisions (1, 2). In a subsequent decision from June 2012, the federal supreme court stated that compulsory treatment was not sufficiently legitimized by any of the existing 16 federal state laws, or by the federal guardianship law that allows hospitalization for a variety of social and health reasons (3). This decision created a legal vacuum, which only allowed enforced medication in terms of emergency treatment, legitimized by a state of immediate emergency (§ 34 StGB). However, emergency treatment is restricted to a single treatment in an acute life-threatening crisis. After a reform of the guardianship law in February 2013 and of the Mental Health Laws (PsychKHGs) of the 16 federal states between 2015 and 2018, compulsory treatment in patients with lack of insight into their illness is permitted again but only after judicial approval with strict procedural requirements (e.g., after a distinct court decision, which is based on the expertise of an independent psychiatrist; if there is a danger to the patient’s or others’ health or life, after considerable efforts to persuade the patient to have treatment have failed) (4).

This temporary legal gap provided us with a quasi-experimental situation in which to study how all different actors involved were able to cope with drug refusal in the ward when the option of coercive medication treatment was no longer available. Quantitative analyses of routine data showed that during that period, the number of aggressive incidents as well as the use of seclusion and restraint increased. After the new legislation had come into force, the levels dropped to the level before the ban of involuntary medication (5). Chart analyses of patients who were treated in the period before and during the ban showed that during the ban, there were more restrictions of freedom, while dosages of antipsychotic medication at discharge and the small percentage of those who did not take any medication at discharge remained stable (6).

These results indicate that the actors involved had to deal with significant changes and challenges at that time. Patients may have enjoyed the freedom to simply refuse the offered medication without the risk of involuntary medication. Doctors and nurses may have had to give up the usual routines and find other ways to get patients to take the prescribed medications. Caregivers may have found it confusing to see their family member being kept in the ward without receiving any medication.

Thus, the aim of this study was to explore how representatives of each of these four groups experienced the refusal of medication when the option of involuntary medication was not available anymore. How did they conceptualize their situation? What were the main challenges reported, and what opportunities were seen? How did the actors react? How did they interact with others? Which conflicts arose? Which solutions were found? We chose a qualitative approach in order to explore and to collate the varying perspectives of our respondents, and we deliberately allowed a broad focus in the narratives. In particular, we aimed at finding starting points for reconciling the positions in the antagonism between the patients’ right to self-determination and the professional commitment to avert damage from the patients.

## Materials and Methods

### Participants

Four groups of participants were chosen for the interviews: patients who currently or previously refused antipsychotic medication during inpatient treatment, family members of patients who actually or previously had refused medication in inpatient treatment, and finally, physicians and nursing staff who had experiences with patients who refused medication. The selection of interviewees was guided by the assumption that these groups were affected the most by the changed legal framework.

With one exception, all patients included in the study were currently in inpatient treatment. They had to be diagnosed with schizophrenia or schizoaffective disorder (ICD-10: F20.x or F25.x) or bipolar disorder (ICD-10: F31.x) (7). Besides organic mental disorders, these are the diagnostic groups most often involved in compulsory medication treatment (8). The participants had to have sufficient cognitive abilities and German language skills to be able to participate in the interviews. Participating hospital staff had to be experienced in patients who had been subjected to involuntary treatment, i.e., nearly all of these interviewees were working at psychiatric acute units. The interviewed family members did not have to be related to participating patients, although some actually were. In an extensive preliminary conversation, we verified that all participants understood the procedures and aims of the study.

The search for eligible participants took place according to the snowball principle. Patients were first contacted by the ward staff, who looked for eligible patients meeting the inclusion criteria. They were asked for permission to be contacted by the interviewer. Only then did the interviewer contact them, explain the study, and ask them to give informed consent to participate in the study. The participating hospital staff was addressed directly by the interviewer, or a contact was arranged by colleagues. Family members were approached only after their ill relatives in the ward agreed, or they were addressed directly at meetings of self-help groups.

We intended to use a strategy of purposeful sampling. This approach is widely used in qualitative research in order to get the broadest possible views on an issue while the number of interviews is limited (9, 10). This means we were looking for participants who were experienced in the phenomenon to be investigated (medication refusal) but at the same time represented a variety of experiences within the interviewees’ groups. So, after a few interviews had been conducted, transcribed, screened, and annotated with comments, we reflected on the inherent perspectives of the interviewees. We made up our minds if there were any important aspects missing that had not been covered by the participants. Then we continued the search for eligible participants and added new interviews to the sample. For example, after several interviews with the hospital staff who were critical about the legal changes, we chose to find also some mental health professionals who welcomed the change. After several interviews with patients very critical about their medication, we searched for patients who eventually accepted taking antipsychotics. For this aim, we also interviewed some experts outside the hospital.

### Data Gathering

We conducted guideline-based problem-centered interviews in an open, casual manner (11). The interviews started with a statement explaining the aim of the study: “This study wants to explore your experience with (your relative/patients) refusing to take the medication on ward. I am interested in the circumstances, how it came about, and how you and your environment dealt with the situation. I am also interested in how you evaluate and judge the situation now.” The interviews covered primarily the issues the interviewee wanted to talk about, but the guidelines served as a checklist for relevant topics that had not yet been covered during the course of the interview. The guidelines comprised the following topics: consequences of the juridical situation for the individual; visible implications on structures and processes in the hospital; effects on the relationships between therapists, nurses, patients, and relatives, motives of treatment refusal; handling of the consequences; and suggestions for resolution or improvement of unsatisfactory situations.

All interviews were audio recorded and verbally transcribed. Interviewees were given pseudonyms. The mean length of one interview was 21 min.

### Data Analysis

**Figure 1:**
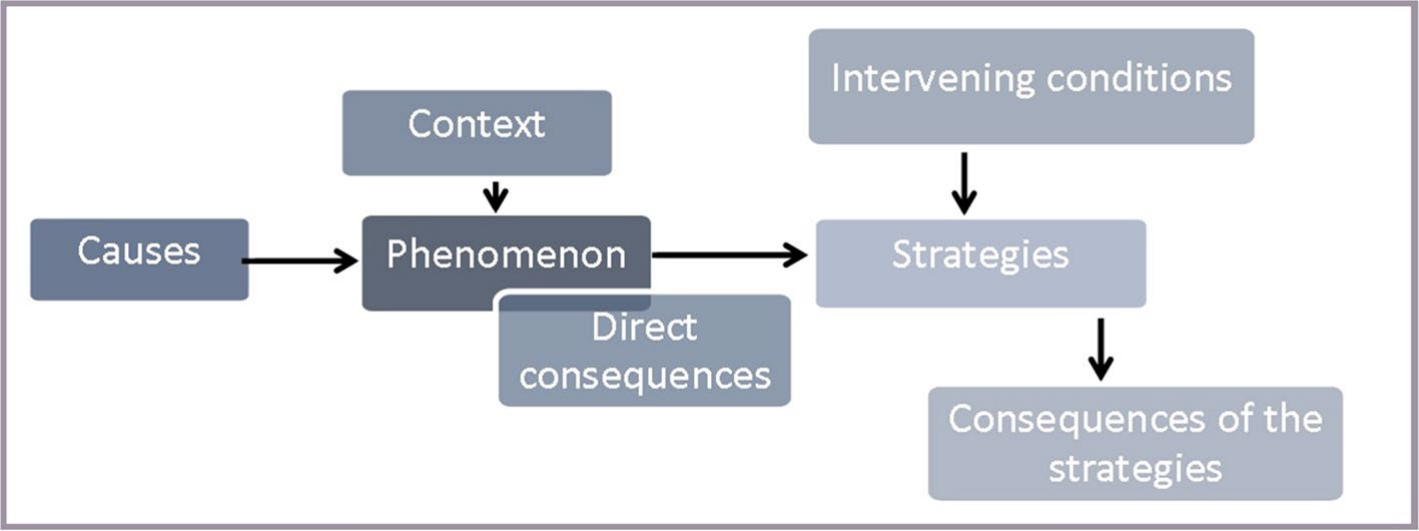
Research paradigm according to Strauss and Corbin (13, p. 78).

We analyzed the interviews in a qualitative manner by using a reference frame of grounded theory methodology (GTM) (12–15). Due to practical reasons, we deviated from strict GTM at some points and modified the method. This relates to the sampling of the participants (for technical reasons, the time of recruitment was limited) and to the restriction of the analysis to a central topic shared by all participating groups. The analytical process was supported and documented by using the software atlas.ti (16). After the open coding, the different groups’ perspectives were conflated in a model based on a paradigm of Strauss and Corbin (13). This paradigm included a central phenomenon, the causes and context of the phenomenon, the direct consequences of the phenomenon, the actions being taken, intervening factors influencing the actions, and the consequences of actions (cf. **Figure 1**). As a result of the heterogeneous interviews, we chose to define the refusal of medication as the central phenomenon. To ensure intersubjectivity, the coding and the analysis were conducted in close cooperation between the involved researchers. Doubtful cases were discussed until a common solution was reached. The process of analysis and the reflections upon it were documented by collection of emails and by research diaries.

### Ethical Aspects

The study started only after the aim of the study and its procedures had been described in detail to the participant and after he or she had given written informed consent. Confidentiality and anonymity was ensured by pseudonymization already during transcription. The study’s design and procedures were approved by the medical ethics committee of Ulm University (appl. no. 44/13).

## Results

### Participants

Eleven patients participated in the study. Their mean age was 43 years (25 to 60 years), six were male, 82% had a schizophrenia spectrum disorder, and 18% had an affective disorder. They had an average of 10 hospitalizations (1 to 30). The eight participating family members had a mean age of 44 years (19 to 72 years), three were male, and five were female. Their roles were father (two), mother (one), sister (one), spouse (two), and daughter (two). The seven nurses had a mean age of 40 (26 to 49 years), five were male, and two were female. Their professional experience in psychiatry ranged from 3 to 36 years (average, 20 years). The five physicians and one psychologist in a doctor’s role were all male. Their mean age was 46 (30 to 55 years). Their professional experience ranged from 2 to 29 years.

### Content

For the evaluation of the overall results, the verbal content of the individual elements of the research paradigm (e.g., “causes”) is compared group by group.

An overview of all findings is provided in **Table 1** ^1^.

**Table 1:** Overview of results.

	Patients	Family caregivers	Nursing staff	Doctors and therapists
**Phenomenon**	Refusal or discontinuation of medication or complete refusal of treatment	Refusal or discontinuation of medication or complete refusal of treatment	Refusal or discontinuation of medication or complete refusal of treatment	Refusal or discontinuation of medication or complete refusal of treatment
**Reasons**	Striving for autonomy Conceptualization of the problem and of recovery Medication attitudes and experiences Stigma of mental illness	Lack of insight into illness and need of treatment Negative experiences with mental health care Side effects of medication Living circumstances	Striving for autonomy Lack of insight into illness and need of treatment Side effects of medication Mistrust and allegations against the mental health care system	Lack of insight into illness and need of treatment Lack of insight into the consequences of no treatment Positive experience of symptoms Mistrust and allegations against the mental health care system
**Consequences of phenomenon**	Negative consequences of lack of medication (self/others)→ symptoms/conflicts Negative consequences of reaction of others → fear and coercion Positive consequences → feeling good	Negative consequences for the patient → deterioration of symptoms, conflicts, use of coercion Negative consequences for family → stress, alienation, deterioration of relationship, frustration, resignation Negative consequences for doctor → helplessness	Negative long- and short-term consequences for the patient (from deterioration of symptoms to social decline and impaired societal participation) Negative consequences for the environment (family, nursing staff, doctors, fellow patients) Negative consequences for ward atmosphere	Negative long- and short-term consequences (from deterioration of symptoms to social decline) Negative consequences for the family
**Conceptualization of problem**	Involuntary hospitalization Experience of coercive measures (present and past) Fear of impending coercive measures and of being threatened to take medication	Own feelings of helplessness, burden of illness history to the family Negative consequences of refusal of professional treatment by ill family member, especially refusal of medication New burden to the family due to changed legal situation that affects the options in inpatient treatment	Involuntary inpatients needing adequate treatment but refusing to take medication Changed legal situation limiting one’s options of professional acting and thus conflicting with understanding of one’s professional role and ethics Restrictions in action options leading to conflicts in the ward and to an increase in workload (management of noise, danger, conflicts, etc).	Involuntary inpatients needing adequate treatment but refusing to take medication Changed legal situation limiting one’s options of professional acting and thus conflicting with understanding of one’s professional role and ethics—opportunity for a change Keeping untreated inpatients on a ward leading to conflicts with fellow patients, employees, and relatives
**Goals**	Escape the other-directed situation and decide about one’s own life again and about how to deal with the illness	Help the family member Get help and find relief for oneself	Provide the patient with adequate treatment to relieve suffering Maintain smooth ward management to make all inpatients feel safe and recover	Provide the patient with adequate treatment to relieve suffering, prevent deterioration, and restore the capacity for self-determination Have available all the necessary measures to do the job in a professionally and ethical way
**Strategies**	Cooperative strategies (talking, negotiation, yielding) Confrontational strategies (refusal, insistence, protest rebellion, legal action) Learning to live with and beyond illness Dealing with family caregivers	Coping with illness of the patient (emotionally and cognitively) Coping with stress/self-care Dealing with patient (support vs. control) Seeking and involving professional help → initiation and support of inpatient treatment	Patient-centered strategies (building a relationship, involvement in treatment decisions, individual approaches) Pressure to make the patient take the medication De-escalation Protection of fellow patients Coercive measures Efforts in increasing team cooperation Leaving the patient untreated (increased attention to the need of intervention) Involvement of family members in treatment	Patient-centered strategies (building a trustful relationship, information, negotiation, shared decision-making approaches…) Medication management (balancing risks and benefits of no medication, management of side effects, accompanied discontinuation) Alternatives to medication treatment Alternatives to coercion Improvement of framing conditions in the ward and in the hospital (e.g., rooms, atmosphere, staffing) Involuntary medication Discharge without medication treatment Involvement of family members in treatment
**Influential factors**	Acceptance of (involuntary) hospitalization Acceptance of medication treatment Former experiences with coercion and medication Relationship with family Relationship with doctor and clinical staff Personality/character	Conceptualization of and experience with illness Knowledge and acceptance of inpatient treatment Experience with and attitudes towards involuntary hospitalization Attitudes towards medication Trust in professional competence Experiences in the psychiatric ward Emotions Economic resources Relationship dynamics within the family Perception of insight and responsiveness of patient	Condition of the patient, character, previous experiences with patient Trustful relationship between patient and staff Personal and professional competences and attitudes of staff members (e.g., de-escalation) Resources at the workplace (e.g., staffing, space) Professional and individual attitudes towards use of coercive measures Ward atmosphere/ward policy Hospital policy	Condition of the patient, previous experiences Society (role expectations, image of mental illness) Attitudes towards use of medication Understanding of their own professional role and professional ethics
**Consequences of strategies**	Cooperative strategies (taking medication) → compromises achieved, less conflict within family, positive and negative side effects of medication Confrontational strategies → no treatment, experience of coercion in the ward, success in court Not being allowed to leave the hospital → frustration and disappointment Being subjected to coercive measures → frustration, anger, fear; might have been justified; might have been stressful for professionals	Seeking professional help → relief and hope vs. feeling rejected Pressure and control → mistrust in patient, conflicts vs. patient gives in without conviction Efforts to engage the patient in therapy → successful vs. disappointment when repeatedly unsuccessful Support and protection → sometimes stress, sometimes patient rejects support, improvement of understanding between patient and staff	Patient-centered approaches → often successful in engaging patient in treatment Use of pressure → sometimes successful in promoting cooperation, often not sustainable Use of seclusion/mechanical restraint → stressful for staff and patients, harm to therapeutic relationship, subsequent cooperation in some, protection of others Use of involuntary medication → stressful for staff and patients, humiliating, harm to relationship vs. improvement of symptoms Leaving the patient without treatment → stress in staff, patient, fellow patients, and family members	Patient-centered approaches → successful, though time-consuming Use of pressure and coercion → positive and negative effects (improvement of symptoms, restoring ability to communicate vs. traumatization and harm of trust and relationship) Leaving patient untreated due to legal restrictions → no improvement, deterioration, subsequent harm to others, allegations of some patients; conflicting with own professional and ethical norms; frustration and anger in family caregivers and nursing staff
**Others**	Mental health professionals → Disrespect patients’ autonomy → frustration, powerlessness, humiliation, trauma → Respect patients’ autonomy → negotiation → compromise/solutions *Dependent on* → Attitudes towards medication → Understanding of professional role → Interest in patient Family → Pressure → conflicts, blame, mistrust, sometimes (retrospectively) thankfulness → Support → improvement of relationship, thankfulness *Dependent on* → Trust and respect within family (support or control)	Patient → Cooperates → hope vs. doubts → Does not cooperate → disappointment *Dependent on* → Willingness of patient to receive treatment → Inner familiar relationship and trust → Patient’s character Doctors and nursing staff → Pressure/coercion → promotes short-term compliance, long-term cooperation questionable, danger of traumatization → Persistence in convincing the patient to engage in treatment → successful → Discharge without treatment → burden for families → Involvement of family vs. too little involvement → relief and increase in knowledge vs. helplessness, feeling rejected, disappointment *Dependent on* → Restrictive legal frame → helplessness in professionals	Patients → Give in and take medication → symptoms improve → Take their rights to refuse/take legal action → enjoy their increased power → negative consequences for ward atmosphere *Dependent on* → Insight and willingness to receive treatment → Trust and relationship with caregivers → Knowledge of legal situation Doctors → Weigh the risk of involuntary medication against the risk of leaving without medication → Take legal action → Involve family members in treatment *Dependent on* → Therapeutic relationship → Changed legal frame Family members → Reproaches to the ward when patient is discharged prematurely → staff feel misunderstood *Dependent on* → Expectations in the hospital to cure the patient	Patients → Negotiate persistently but give in → improvement in symptoms, relief for others → Motivate other patients to refuse medication → negative consequences for ward atmosphere → Take legal actions → Are burdensome for their families *Dependent on* → Willingness to receiving treatment/insight/experiences → Current symptoms and perception of situation → Family members do not set limits Nursing staff → Individual patient-centered approaches to the patient → successful in building of trust *Dependent on* → Individual and professional competences → Previous experiences with patient Family members → Not setting limits to patient → conflicts in the family → deterioration of disorder

#### Central Phenomenon, Reasons, and Motives

All interviewees of the four groups were asked about the refusal of medication. Accordingly, we determined this issue as the central (common) phenomenon of the analysis and as the starting point of an action model according to the work of Strauss and Corbin (13). The use of an action model was helpful to disclose motives, reasons, consequences, actions, intervening conditions that shaped the actions, and finally, the consequences of actions. The narratives developed around this central element, some in a similar story line, some in rather divergent narratives. In all groups, the phenomenon “refusal of medication” was described and constructed in a similar way. It could include a mere “no” to medication but also an irregular use of the medication (e.g., taking less than prescribed or not taking it every day), cheating when taking the medication, or taking it involuntarily only in reaction to strong external pressure.

**Figure 2:**
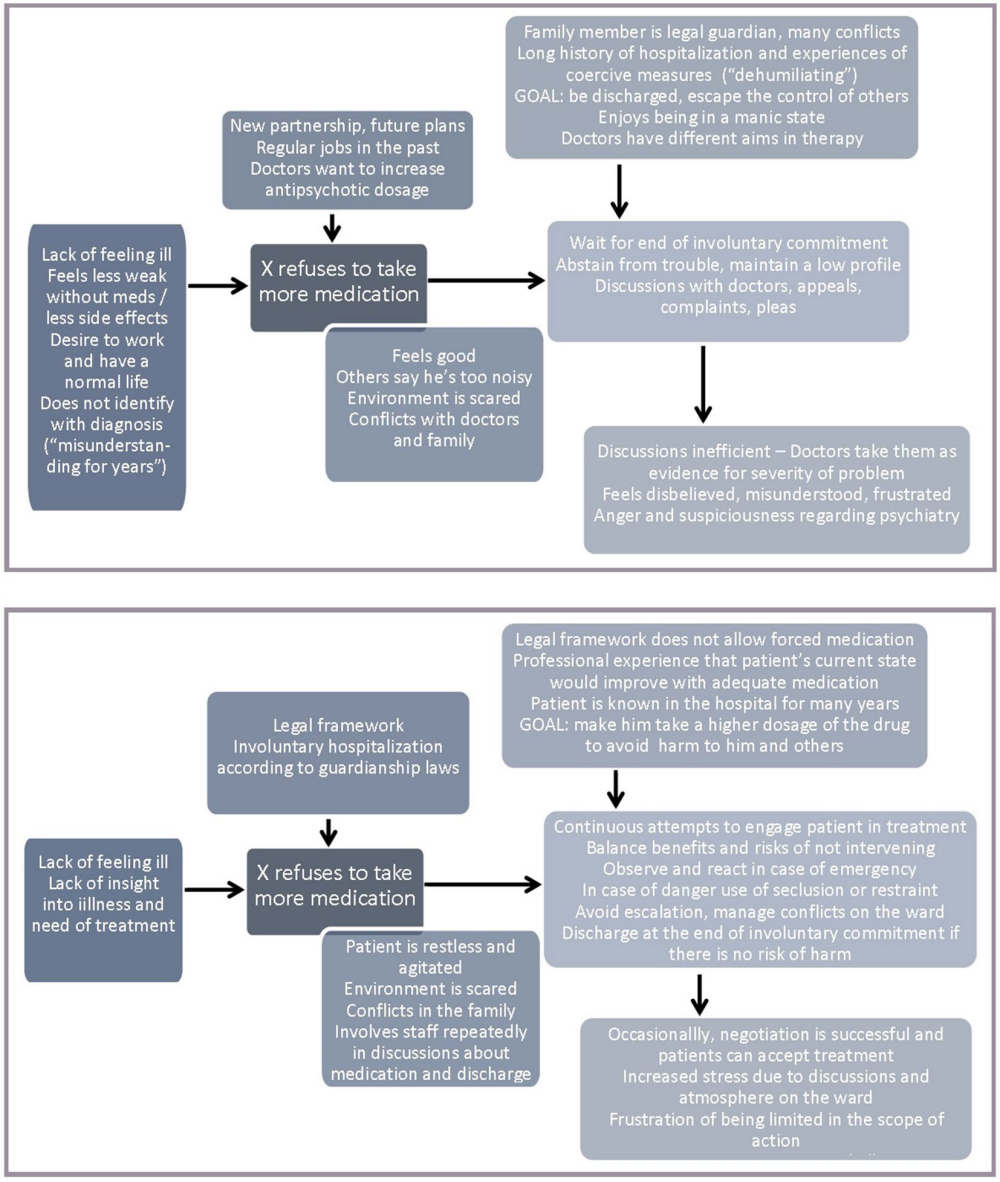
Two typical complementary examples: patient (above) and doctor (below).

**Figure 2** shows two typical, complementary examples of the analyses, one of a patient who refuses to take a higher dosage of his medication and one of a doctor dealing with such a situation. In this example, a vicious circle arises when the patient’s actions (e.g., discussions with doctors) to defend his interests (e.g., avoid being prescribed a higher dosage, enjoy new partnership without feeling restricted by medication) are regarded as a direct consequence of the phenomenon by the professionals (e.g., increased symptoms such as agitation) and as evidence for the need to increase the efforts to make the patient take more medication in order to relax. These efforts are taken as evidence by the patient that professionals rarely listen to his needs and just want to tranquillize him.

“I just want to live again without this handbrake” (Franz, patient)

The main motives for refusal according to patients and family members were different conceptualizations of what was the problem to be treated. Some patients did not think they had a problem at all. Some thought they had other problems and medication treatment was not the adequate therapy. They did not feel understood by professional helpers. These narratives were substantiated by memories of being subjected to coercion and negative experiences with former inpatient treatment, drug treatment, and encounters with therapists. Family members reported similar observations. Patients and family members also talked about impairments of functioning, which they attributed to the medication. Some interviewees not only blamed antipsychotics but also were negative about the use of chemical substances in general. Drug attitudes included the fear of stigmatization by being considered mentally ill because they were taking medication. They also included a general distrust of the benefits of medication, and sometimes, there was a desire not to interfere with the pleasant effects of the condition by taking antipsychotics. On the patients’ side, there was a strong desire to make decisions about one’s own health independently and not allow others to dictate them.

“Many claim that they were primarily made ill by psychiatry” (Heiner, doctor)

The views of the interviewed nursing staff and the doctors were consistent with these explanations of medication refusal. However, in their understanding, the patients’ different definition of the problem or their doubts as to whether they needed to be treated at all was part of the concept “lack of insight into the illness”. In the eyes of the professionals, this was associated with an impairment of insight into the long-term consequences of an untreated psychotic disorder. According to the interviewees, another reason for noncompliance was patients’ distrust, often in response to previous negative experiences with the health care system. Some interviewees said that sometimes they (or the hospital or the medication) were held responsible for an interrupted or completely destroyed biography. Medication would have made them sick. Accordingly, patients would not want to take it. All professionals also mentioned the changed legal situation. Some said it would encourage the patients’ ambitions to defend their autonomy by refusing drug treatment.

#### Consequences of Medication Refusal

“So my mother kept pointing this out to me, take the meds, take the meds, I have not taken them often, there was also quarrel in the family about it…” (Jean-Jacques, patient)

Interviewees in all four groups similarly reported negative consequences of medication refusal or discontinuation. Positive consequences were rarely reported. A few patients claimed to feel more energetic and alive without medication. But even these patients told of negative consequences. They were more often involved in conflicts with others, and others were afraid or did not understand them. In addition, interviewed patients felt at constant risk of being involuntarily hospitalized and subjected to other coercive measures that some had previously experienced. Many patients reported extensively on previous experiences with compulsive medication. They found it humiliating and were worried that this might happen again.

“…they simply do not function properly out there, they do not get along with their family, their social structures break, they lose their flat, lose their job…” (Andy, doctor)

Apart from the deterioration of the patient’s condition, professionals and family members mentioned the risk of harmful long-term consequences or social decline. Persisting symptoms due to refusal of treatment also had an impact on the social environment of patients, especially with regard to family and friends (loss of confidence, conflicts, worries, resignation, hopelessness, and fears of the future). This was also noticed by doctors and staff in the ward. They reported that the refusal of medication led to longer hospital stays of patients with untreated symptoms. This not only complicated the work of health care professionals but also affected the interactions in the ward, the teams, and fellow patients.

#### Focus, Strategies, and Consequences

“But, what’s outside, when I’m back at my family doctor’s, that’s different” (Gertrude, patient)

As actors of the four perspectives had different focuses and different interests, they were also using different strategies and actions to cope with the situation. The patients referred to the involuntary hospitalization, the compulsion to take medications, and the threat of being treated against their will as their main problems. Accordingly, one of their priorities was the question of how to withstand the pressure and escape the foreign-controlled situation. Many actions related to how patients managed to continue to refuse medication and how the staff tried to make them give in. Patients talked about their struggles, arguments, and discussions with doctors, nurses, and relatives to convince them that they needed other treatment, if any (e.g., more talk). Some reported that they had finally given up and taken the medicine, although they were not convinced of the medical benefit. They did it only to avoid the risk of being involuntarily drugged, to increase the possibility of early discharge from the hospital, and to escape the control of others.

All of the interviewed patients were involved in an individual process of weighing costs (e.g., medication side effects or general discomfort with drugs) and benefits (e.g., regaining autonomy after discharge). Some even said that now they were certain that they needed antipsychotic drug therapy to prevent relapse. Finding a way to deal with the disease without making it the center of one’s own life has been considered by some as an important step in achieving an inner arrangement. Another line of conflict related to dealing with family members. The patients often followed various strategies to keep their distance so as not to be controlled. This, in turn, was noted by relatives and increased their sense of losing control of the situation, the sadness of losing touch with the patient, mistrust, anger, and worry about the future.

“I just wish that everything goes well, and hope and trust that someday his insight will come” (Peter, father)

Most of the relatives described feelings of helplessness and talked about their burden of disease, which they often had faced for years without the prospect of change for the better. On the one hand, they reported on how they had tried to support their ill family member for a long time in coping with everyday life and with the disease, through close control or by supporting the patient’s desire for independence. On the other hand, the experience of powerlessness was omnipresent.

Families talked about their efforts to engage the patient in treatment, through pressure or empathy and gentle persuasion. They sought help in the professional system. Some initiated involuntary hospitalization and then felt insecure and bad about this step. Following the admission of their family member to the hospital, they tried to stay in touch with doctors or to participate in treatment decisions, with mixed results. Some felt rejected by the professionals, and they missed more detailed information about the treatment process, mainly due to a lack of time. Others took on a mediating role between their family member who refused to speak and the professionals by translating the perspectives of each side to the other, in order to strengthen cooperation between the two. Some families advocated coercive measures and compulsory medication to get the patient into treatment. At the same time, it hurt them, and they felt uncomfortable. An important issue for almost all family members was their own coping with the disease and the current tense situation, and how they tried to take care of themselves (e.g., trying to remember the “real” person with a biography behind the alienated son or daughter, trying to find a healthy distance, seeking support for oneself, struggling for an inner acceptance of the situation, trying to get information and make sense of the disease).

“This depends entirely on the patient, but the fact that you can treat later will ultimately delay the entire treatment. It takes longer in the end.” (Patricia, nurse)

From the doctors’ and nurses’ perspective, the problem had a different focus: There was a patient in the ward who refused the type of treatment that, according to professional experience, would likely help him or her recover. Due to the changed legal situation, involuntary medication could only be used in an emergency. This led to a conflict between the self-conception of the professional role and the legal restrictions to do what is necessary for medical and ethical reasons. Moreover, not only the involuntary patient with severe symptoms was affected. The staff reported that the situation had an impact on the other patients and on the ward atmosphere in general. On the one hand, the nursing staff tried to increase the patient’s motivation to take the medications offered, which included building trust and reducing fears and concerns, and on the other hand, they tried to manage the ward in such a way that the other patients were not too affected and could recover.

The interviewees described their ongoing efforts to individually address the patient, establish good relationships, build trust, and seek a shared solution. Most of the time, such an individual approach proved successful. Furthermore, the staff members explained their strategies to resolve tense situations at the ward, calm down agitated patients, and de-escalate conflicts. They reported on trainings in these practices. They also talked about situations where de-escalation did not work anymore. They attempted to avert immediate harm from the other patients or the agitated patient by using coercive measures such as mechanical restraint or seclusion. These procedures follow a strict routine familiar to every staff member. It was reported that coercive measures were stressful for all participants involved, such as staff members and patients, and there was a general commitment to avoid them. However, some interviewees stated that the experience of coercion was sometimes the starting point for the person concerned to finally allow medication treatment. Good cooperation within the team was mentioned by the members to ensure good and responsible ward management and treatment. Transparent and clear communication within the team was a crucial prerequisite to remain able to act and to avoid team conflicts under difficult working conditions. Although this has always been important, the new challenges of the changed legal situation, in which the use of involuntary medication was very limited, made it even more significant.

“…and after a few weeks or maybe even after a few months of tough negotiation and staying with it, in almost all cases a consensual treatment planning has emerged” (Ferdinand, doctor)

As mentioned above, physicians faced the same dilemma as nurses—patients who (according to professional knowledge and experience) would benefit from the medication decidedly refused it, and the legal restrictions made it almost impossible to use the necessary drugs against the patient’s will. The interviewed physicians reported their efforts to convince patients of drug treatment through various means, ranging from verbal pressure to negotiation, patient-centered communication, and shared decision-making. Preconditions were efforts to build trust and establish a sustainable working relationship. As the nurses had already reported, increased efforts to address individuals individually were considered helpful and promising. However, interviewees pointed out that these procedures were resource-intensive and time-consuming, time that would have to be taken from other patients. Thus, the interviewed physicians described how they weighed the benefits and risks of leaving a patient unmedicated for at least some time, and confined themselves to monitoring the patient more closely in order to intervene immediately in case of deterioration. Some doctors and nurses said it gave them a hard time to wait for an emergency that would finally legitimize the application of the appropriate medication. They all had in mind that it was only one year earlier that there was no doubt about doing what the situation would have required.

When there were no more reasons to keep an untreated patient in the ward involuntarily, doctors sometimes decided to discharge him or her. This, in turn, was a major challenge for relatives who had hoped to get help for their family member and hand over responsibility to the hospital for some time. It was an unsatisfactory and frustrating solution for the doctors, as they expected no improvement in the patient’s symptoms without treatment. They pointed to the risk of chronification and increasing functional impairments. The use of involuntary medication was limited to emergencies. In some cases, the doctors said they had tried to obtain juridical permission to use it. They saw the risk of traumatization as a result of coercive measures restricting freedom, such as seclusion or restraint, which could negatively impact the future therapeutic relationship, the willingness to consider medical advice, and future help-seeking behavior. However, there was also the experience that in some patients, the use of involuntary medication was the beginning of a successful drug treatment.

#### Influencing Factors

“It has been the purest horror for me, always been, the idea I would have to go back in there, and so I have clearly said to my sister, do not bring me to psychiatry” (Elvis, patient)

The most important influencing factors for the patients were past and present experiences with the mental health care system, experiences with the voluntariness of the admission and the stay, encounters with the actors involved in the admission, encounters in the ward, previous experiences with therapy procedures, and the current therapy offers. Moreover, the acceptance of inpatient treatment and attitudes towards medication (antipsychotics, but also drugs in general) had an impact on how patients responded to attempts to engage them in medication treatment. Other important influential factors were confidence in the competence of the doctor, the impression of being understood, and sympathy. Some patients also attributed their behavior to their individual character. While the interviewees of the other groups described it as “lack of insight,” patients who refused to take the medication seemed to be absolutely sure of their decision, even more so since they believed that their problem was rooted in their biography and was different from what the doctors claimed. However, there were also some patients who in retrospect said that now they knew they needed medication to get better.

“No, but she is no longer herself in that point. I quite understand the whole thing as an illness. I know what she was like before.” (Rose, sister)

According to hospital staff and family members, the ability to empathize, understanding, and the quality of the relationship were key factors in interacting with each patient. A major influence on most family members was the experience of a long history of repeated illness episodes with varying degrees of hope and frustration. The actions of family members were often determined by their emotions such as compassion, concern, or anger. But also cognitive factors such as mental health literacy, illness concepts and concepts of recovery, knowledge about the drug treatment, and their own attitude to the use of medication had an impact. Trust in the expertise of the mental health professionals, as well as experiences with inpatient treatment and the impression of the ward atmosphere, also influenced how relatives made efforts or supported others’ efforts to convince the patient of the need to take medication. In some cases, economic issues such as financial options also played a role. Some parents reported that their children’s economic dependency was an efficient leverage to make them engage in treatment.

“But this fundamental paternalistic attitude, that is, the idea of my right to treatment is above the personality right of the patient, of course you can’t do that” (Angie, doctor)

The interviewed professionals added to the abovementioned factors the actual condition of the patient and the assessment of current and future risk of harm. Nearly all respondents talked about their understanding of their professional roles, role expectations of society, professional ethics, and their professional experience, which guided their behavior and their ideas of what to do. Each clinical practice guideline recommends that patients with a psychotic disorder should be provided with adequate antipsychotic medication. Withholding this therapy would therefore be against good practice.

However, they considered the changes in the legal framework as limiting their options to treat patients appropriately (if necessary, even against the patients’ will). At the same time, the change was seen by some interviewees as a catalyst to reflect on the use of coercion in inpatient treatment and to question paternalism-driven long-standing clinical practices and attitudes in treatment. According to the respondents, it has encouraged efforts to improve communication, interaction, and negotiation with patients, aiming for a viable solution and a common decision on the right treatment. Strategies and actions were thus influenced not only by individual and professional attitudes but also by political and societal developments. In addition, some interviewees identified workplace conditions (sufficient staff, ward composition and occupancy, space, ward spirit, etc).

#### Perception of Others

“I often get the impression that psychiatrists think drugs regulate everything…” (Jean-Jacques, patient)

The interviewed patients presented many examples of how they felt disrespected during inpatient treatment. Disrespect was perceived when doctors or nurses demonstrated authoritarian behavior, verbal pressure, etc. Part of the problem, according to the patients, was that mental health professionals seemed to be so strongly committed to using medication to treat mental health problems that they would not listen to the patients. However, many patients also recalled other interactions with dedicated physicians who took their time to explain and negotiate therapy options. This made the patients feel respected and accept recommendations more easily. According to the interviewees, it depends on the attitude of the doctor, the understanding of the professional role, and the individual interest to comprehend the needs of a patient holistically. With respect to their relatives, some interviewees reported how they felt supported, and others how they felt controlled and under pressure. Some believed that they were seeing some kind of coalition or conspiracy between their parents and the doctors, leading to mistrust and secrecy. Some patients assumed that their relatives’ behavior was motivated by their intention to help. Others suspected ambitions to dominate the patient, or lack of understanding in their family.

“Nah, my God, just a little bit of explanation. How to help, or what to do.” (Antonio, spouse)

The interviewed family members talked in detail about cooperative or less cooperative actions of their ill relative, which they often attributed to his or her character, personality, and biography. Often the stories went back to the childhood of the patients. Nearly all interviewees related current behavior to the patient’s history and previous negative experiences with physicians, with inpatient treatment, and with coercive measures. There was a strong desire to examine and understand what had gone wrong in the past and to understand the meaning of the disorder. Some interviewees reflected on their family relationships. They suspected that this also played a role in the behavior of their ill family member. They talked about continued rejection and mistrust of the patient, and how much they were affected. Moreover, they felt ashamed and disappointed with regard to some of the patient’s behavior, and they were mostly worried about the future.

With regard to the professionals’ actions and strategies to get the patient engaged in treatment, the family members had made varying observations—staff members who were very committed and interacted empathetically with the patient and staff members who were overworked and not responsive. As a consequence of the changed legal situation, some patients had been discharged prematurely when they were no longer at risk of harming themselves or others. The families found this an additional burden, especially as they had hoped to hand over responsibility and get help. A father concluded that obviously, the doctors were powerless, too. In addition, many family members complained that they were not involved in therapy decisions. Some missed getting a basic understanding about the disorder, the treatment, and the proceedings in the ward. They reported their experience that doctors or staff did not have enough time to talk to them and respond to their questions and concerns. The use of coercive measures was perceived as a double-edged sword. On the one hand, family members noted a short-term improvement. On the other hand, they reported that the effect was unsustainable and had not changed the insight, and because of this experience, patients were even more negative regarding compliance with treatment recommendations.

“Then it’s hard for the relatives, because they actually bring them to get them cured so they can live outside” (Johanna, nurse)

Health care professionals talked mainly about the changed legal situation and its consequences for inpatient care, ward management, and ward atmosphere. Agitated, involuntarily hospitalized patients who resisted treatment were perceived as a major challenge for ward management. The recovery of other inpatients was affected by an excited and restless ward atmosphere. Many discussions and the need for permanent observation and spontaneous intervention to avert harm had resulted in exhaustion of personal and professional capacities. In both groups, doctors and nursing staff, some interviewees also talked of the burden on family caregivers. Some reported that they were accused of not doing enough, e.g., discharging the patient prematurely. For the interviewees, this was a frustrating situation. One nurse put it this way: “They just do not understand that our hands are tied.”

#### Perception of the Legal Situation

“And the flip side of the matter is, so to speak, that nobody cares anymore” (Amanda, doctor)

Few of the interviewed patients had even noticed that the legal situation had changed. Most said they had not heard of it, or they said they did not care. However, some of the family members said they had been informed by the nurses or doctors. The prospect that their relatives could not be treated according to the state of the art or might even be discharged prematurely without adequate treatment made them feel desperate, helpless, and alone.

Most nurses reported how much the legal situation had changed their daily work, i.e., more violent incidents, an increase of seclusion and restraint, the loss of an important lever to increase motivation to take medication, conflicts in the teams, and an overall increase of workload. Ward management and efforts to ensure safe conditions for everyone took up a lot of resources. In their opinion, the loss of the option of involuntary medication ultimately harmed the patients and their families. Nevertheless, some nurses were positive about the situation. They saw this as an opportunity for overdue critical reflection on coercive practices, routines, and attitudes in contemporary psychiatry—everyone was literally called upon to strive for more focus on the individual patient and had to be creative in getting patients to cooperate in treatment. In some cases, however, legal certainty and new laws would be required to allow coercive medication under certain conditions to increase the scope of action.

The doctors reported the same problems with ward management as the nurses. The ward atmosphere and the working conditions were impaired. There was much frustration about being hindered from treating patients who obviously needed medication to get better. They emphasized the negative consequences of the temporary ban on involuntary medication for their patients, their families, and society. But in this group, too, we gathered voices that welcomed the situation as a decisive push to overcome paternalistic structures and routines in psychiatry.

## Discussion

The study was conducted under very unique conditions, namely, the unusual legislative framework for involuntary treatment. Patients could refuse treatment despite being involuntarily hospitalized. Our aim was to explore by the means of a qualitative analysis how representatives of each of the four involved groups experienced the refusal of medication under these conditions, what kind of problems they were facing, and which solutions emerged. In this special situation, we had also implicitly hoped to learn about alternative reactions to the well-known problem of medication refusal.

We can summarize three main findings: 1) The change in the legislative framework was perceived completely differently and had a different significance in the four groups. 2) The patients’ and family members’ views on medication refusal during involuntary hospitalization were characterized by a biographical, individual perspective. In turn, doctors and nurses shared a professional, medical, and situational perspective. The divergence of perspectives had an impact on problem definition, goals, and solutions. It was a serious obstacle to mutual understanding. 3) According to the interviewees’ reports, continued efforts to address the patient individually, to improve the relationship, and to have respectful communication on equal terms might contribute to make the patient engage in treatment and to avoid escalation in the ward—at least in some cases. On an organizational level, professionals were positive about questioning and rethinking coercive practices in psychiatry, but they also hoped for a timely revision of the legal framework, allowing a wider scope of action again.

We were surprised to learn that only a few of the interviewed patients had even realized that they could refuse medication treatment without having to worry about forced medication. This was in contrast to concerns of the mental health professionals that some patients actually abused their new liberties. The real concerns of the patients were related to the involuntary hospitalization, the associated restrictions, and how to regain control of their own lives. It is known that the perception of coercion is higher in involuntarily than in most voluntarily hospitalized patients (18), and that it is of particular importance in involuntarily hospitalized inpatients to stay in control and maintain a sense of autonomy (19, 20). As expressed by our respondents, too, perceived loss of autonomy went hand in hand with a more negative relationship between the patients and the clinicians (21). Often enough, along with the situation of involuntariness, unpleasant memories of previous hospitalizations and coercion experiences emerged (22, 23). During the involuntary hospital stay, coercion was a permanent latent menace. The ban on involuntary medication was probably not perceived as a real change, since the other measures like seclusion or restraint could still be used. Family members confirmed the patients’ experiences and concerns, but they also reported how much they had hoped to find help in the hospital. Some advocated for the use of coercion, if necessary. The legal changes only played a role when families were disappointed to hear that professionals felt hog-tied and they feared a deterioration of the patient.

In contrast, nurses’ and doctors’ interviews focused mainly on the consequences of the changed legal framework and the manifold related problems. These included concerns that patients might not recover without medication, problems with ward management due to increased aggression, reflections on societal consequences, and inner conflicts of not being able to practice in accordance with professional values.

In fact, during the ban on involuntary medication, there had been a considerable increase in aggressive behavior and in the use of seclusion and restraint, seclusion in particular. As our respondents already reported, suspension of involuntary medication was compensated for by other coercive measures. After the new legislation was set into practice, their numbers decreased again. The laws allowed for involuntary medication again, however, with strict requirements (5). It is hard to determine the exact decline in involuntary medication, because there are no systematic assessments of the prevalence in Germany before the legal framework had changed. According to estimations in the early 2000s, 2% to 8% of all admissions were affected (24). A study in 32 hospitals in Southern Germany in 2016, i.e., after the change, found that involuntary medication affected 0% to 2.5% of all admissions (median, 0.4%) (25). It is difficult to compare the data across hospitals and countries because the frequency of the use of different coercive measures varies considerably across countries due to different laws and cultural sensitivities (26, 27).

While the doctors’ and nurses’ perspective was focused on exacerbating symptoms and the management of unmedicated patients, the patients and their families experienced the situation within a biographical frame characterized by reports of individual illness history, family background, and previous experiences. There was no evidence that refusing medication was intentionally continued during hospitalization as a consequence of the changed legal situation. There are a multitude of reasons for nonadherence in schizophrenia patients that have been studied so far (28). According to their interview study in patients with schizophrenia, Gibson et al. (29) interpret nonadherence as a kind of patients’ treatment choice in order to live well in response to day-to-day challenges of ordinary living. This provides a good description of how our interviewed patients dealt with the medication. Within the biographical context, it appeared more like the result or style of individual coping with the disorder and of the patients’ definition of the “real” problems that had to be fixed (e.g., trauma, conflicts, depression, no job). Mental health professionals might think of the potential long-term harm of untreated psychosis, particularly in first-episode patients (e.g., 30). However, our interviewees perceived the professionals as being virtually obsessed with medication treatment. Some asked if the doctors had nothing else to offer. Although not elaborated, their expectations of an adequate treatment to their problems were somehow different, and patients felt put off with (in their view) a simplistic solution: pills.

Doctors and nurses, in turn, were striving to act according to professional competences and experiences as well as to professional ethics. To deny a patient a medication that might help and prevent harm is perceived as contradictory to the ethical values of beneficence and non-maleficence (31). In this regard, the concept of “poor insight into illness” is often used as a rationale to override the patient’s will (“if he had some insight, he would see the need for treatment”), and to use involuntary medication to restore insight. It might be favorable for the doctor–patient communication to query the conception of poor insight as an all-or-nothing characteristic or just a symptom. For example, in their comprehensive review, Lysaker et al. conceptualize it “not just as the consequence of a failure to notice a problem, grasp a fact or accept a label, but as a failure to make consensually valid sense of complex and potentially traumatic experiences” (32) (p. 18). This approach suggests that it is not enough to rely on education when dealing with poor insight or hope for natural improvement of insight by medication interventions. What is needed is assistance in a fundamental integration process. Effective treatments might target the metacognitive processes involved in poor insight, i.e., guide people with serious mental illness to reflect and make personal meaning of experiences of mental illness (32).

Finally, the relatives seemed to be caught in the middle. They had a biographical perspective, but they were also caregivers who had acquired some health literacy. They struggled for empathy with the patient but also to keep a healthy distance and learn about the disease. Schizophrenia in a family member is a massive burden on family caregivers (33, 34). Many of our respondents were at the end of their strength, and involuntary hospitalization offered some kind of relief and hope. However, their expectations were only partly met by the professionals, whose limited scope of action led to disappointment and irritation. Similar to other studies, caregivers rated involuntary hospitalization as less invasive than patients (35, 36), although there were also concerns about the right treatment of their family member. As has been described in other studies, a delay in receiving help resulted in conflicting emotions and frustration (37). The proceedings in the ward were often perceived as intransparent, especially when professionals apparently did not take the time for communication. The changed legal conditions challenged the families’ confidence in institutional help even more, in particular when patients were discharged without medication. In those cases, even when they expressed their understanding of the doctors, the family members felt powerless and pushed around without a say.

The interview study in this specific setting highlighted the increased pressure on staff members and families when the usual routines had become inoperative. With regard to discovering new practices and solutions in this changed situation to avoid coercion, our success was as limited. The respondents referred to practices to promote de-escalation that are already described in literature, such as efforts to calm down the patient by communication, increase trust, and establish a working relationship (38–40). Patients indicated they were more likely to cooperate in treatment when they had the impression they were taken seriously and when others refrained from authoritarian behavior. In the first place, it might be helpful to acknowledge the patients’ different perspectives and their conceptualization of the situation at each point in the coercive process (41). According to other studies, successful communication with inpatients is supported by a focus on the patient’s concerns, positive regard and personal respect, appropriate involvement of patients in decision-making, genuineness with a personal touch, and the use of a psychological treatment model (42). These ingredients for improvement of cooperation were also reported in our interviews with nurses and doctors. Even though the loss of the lever of involuntary treatment was regretted by some of our respondents, it is also perceived as a chance to work on doing the job differently and to engage in the transformation of a psychiatry that can refrain from coercion.

The ban on involuntary medication had caused many discussions on the wards. It might have increased self-reflection, and it definitely challenged the routines, procedures, and attitudes in the mental health care system. As a consequence of the changed legislation in the federal state of Baden-Wuerttemberg, it has been mandatory since 2015 to collect data on coercive measures in psychiatric hospitals and to supply these data to a central register (25). Although coercive measures are still in use in psychiatry, this register allows monitoring possible changes and evaluating interventions. Moreover, there is more transparency for the public. Since former coercive experiences rest in the patients’ memory for a long time, it is indispensable to provide the nursing and medical staff with the necessary guidelines for dealing with coercion and aggression in the least harmful way. One of the newest guidelines includes a systematic collection of possible single or complex interventions that all have proven to be successful in reducing aspects of coercion (38). These are only some steps to improve the situation, and of course, there also has to be a general change in treatment culture. The vast amount of literature and current research on coercion underlines the universal need for a change.

## Limitations

The study has several limitations dealing with methodological issues: We had chosen an approach of theoretical sampling in order to assess as many different aspects of the problem to be investigated as possible. However, due to technical reasons, the sample was mainly recruited in one hospital, and only a few additional voices from the outside were intentionally selected to enrich the sample of views (13). The study would have profited from taking a broader perspective in recruitment, i.e., other hospitals with different working styles or common experiences.

Regarding the patient sample, it has to be noted that our interviewees were seemingly able to give informed consent and understand the aim of the study. In line with Carpenter et al. (43), we saw no reason to exclude people who were impaired by their disorder in some of their capabilities but were perfectly able to understand the study. A few interview partners showed formal thought disorders like perseverations or tangential answers, which impeded the analysis in some texts. However, one crucial shortcoming of the study was the fact that we were not able to talk to patients who were currently deeply absorbed by delusions and distorted perceptions of reality. Of course, these would have been exactly the patients who were the main target of professionals’ intentions of using involuntary medication in order to restore their ability to make responsible decisions.

There are other sampling issues: The majority of the interviewed nurses and all of the interviewed doctors were male. Thus, a specific female professional perspective is definitely missing. Moreover, the very heterogeneous reports in the group of the family members indicated that we did not reach theoretical saturation in this group (13). Besides, for technical reasons, the period for searching for interview partners was limited; thus, we did not include additional interview partners after a primary analysis. This would have been necessary to verify our results by new text content.

Another problem was changes in the legislation during the period of the interviews. From February 2013 on, it was again allowed to use involuntary medication according to the law of guardianship, after juridical approval with high requirements concerning the procedures. Part of the interviews was conducted after this reform. The concerned interviewees reported retrospectively about the situation, and this limits the comparability with the interviews before the change.

We decided to use a research paradigm (13) as a heuristic tool for finding and structuring the multitude of aspects in the texts. As a so-called central phenomenon, we defined the issue that appeared in all interviews: refusal of medication. By this approach, we limited our analysis deliberately to aspects of medication treatment. However, refusing medication had a different significance among the different actors and in the reported chains of action, which is reflected in the results. A different approach for obtaining equally meaningful results might have been the identification of core concepts in the different perspectives (e.g., constructions of recovery).

## Conclusion

The temporary ban of involuntary treatment during inpatient treatment has led to many discussions among practitioners about how to control and manage the situation. Although there were no new solutions to the problem of patients refusing medication treatment, our study shows that it is indispensable to be aware of the fundamentally different perspectives of mental health professionals, inpatients, and family caregivers. Efforts are required to implement collaborative structures and client-centered approaches as well as a critical reflection on usual practices and attitudes, while not losing sight of the burdened families. Reconciliation of the diverging perspectives seems to be difficult but not impossible. It is all about relationships and communication.

## Ethics Statement

The study started only after the aim of the study and its procedures had been described in detail to the participant and after he or she had given written informed consent. Confidentiality and anonymity were ensured by pseudonymization already during transcription. The study’s design and procedures were approved by the medical ethics committee of Ulm University (appl. no. 44/13).

## Author Contributions

SJ, FH, and TS designed the study and wrote the protocol. FH conducted the interviews. SJ and FH undertook the analysis. FH wrote her doctoral thesis on the study. SJ wrote the first draft of the manuscript based on this thesis. All the authors commented on the manuscript. All the authors contributed to and have approved the final manuscript.

## Conflict of Interest Statement

The authors declare that the research was conducted in the absence of any commercial or financial relationships that could be construed as a potential conflict of interest.

## Footnotes

1 An extended version of the results and codes can be found in Hüther et al. (17). The appendix including the codes with verbal examples (in German) can be freely accessed at the URL http://www.psychiatrie-verlag.de/buecher/detail/book-detail/behandlungsverweigerung-patientenautonomie-und-zwangsmedikation-1.html.

## References

[1] BVerfG Beschluss des Zweiten Senats vom 12. Oktober 2011 - 2 BvR 633/11 - Rn. (1-47). http://www.bverfg.de/e/rs20111012_2bvr063311.html.

[2] BVerfG Beschluss des Zweiten Senats vom 23. März 2011 - 2 BvR 882/09 - Rn. (1-83). http://www.bverfg.de/e/rs20110323_2bvr088209.html.

[3] Flammer E Steinert T Auswirkungen der vorübergehend fehlenden Rechtsgrundlage für Zwangsbehandlungen auf die Häufigkeit aggressiver Vorfälle und freiheitseinschränkender mechanischer Zwangsmaßnahmen bei Patienten mit psychotischen Störungen. Psychiatr Prax (2015) 42(5):260–6. 10.1055/s-0034-1370069 24858428

[4] Gerlinger G Deister A Heinz A Koller M Müller S Steinert T Nach der Reform ist vor der Reform: Ergebnisse der Novellierungsprozesse der Psychisch-Kranken-Hilfe-Gesetze der Bundesländer. Nervenarzt (2019) 90(1):45–57. 10.1007/s00115-018-0612-3 30191253

[5] Flammer E Steinert T Association between restriction of involuntary medication and frequency of coercive measures and violent incidents. Psychiatr Serv (2016) 67(12):1315–20. 10.1176/appi.ps.201500476 27476807

[6] Steinert T Keyssner S Schmid P Flammer E Auswirkungen der vorübergehend fehlenden Genehmigungsfähigkeit für Zwangsbehandlung in Baden-Württemberg: nicht weniger Medikamente, aber längere Freiheitsentziehung. Fortschr Neurol Psychiat (2019) 87. (in print). 10.1055/a-0893-6507 31234213

[7] Dilling H Freyberger HJ Taschenführer zur ICD-10-Klassifikation psychischer Störungen Weltgesundheitsorganisation. 7., überarb. Aufl. unter Berücksichtigung der Änderungen entsprechend ICD-10-GM (German Modification) Bern: Huber (2014).

[8] Flammer E Steinert T Involuntary medication, seclusion, and restraint in German psychiatric hospitals after the adoption of legislation in 2013. Front Psychiatry (2015) 6:153. 10.3389/fpsyt.2015.00153 26578985 PMC4623390

[9] Patton MQ Qualitative research & evaluation methods. In: Thousand Oaks., 3. ed Calif.: Sage (2002). Available from: URL: http://www.loc.gov/catdir/enhancements/fy0658/2001005181-d.html.

[10] Palinkas LA Horwitz SM Green CA Wisdom JP Duan N Hoagwood K Purposeful sampling for qualitative data collection and analysis in mixed method implementation research. Adm Policy Ment Health (2015) 42(5):533–44. 10.1007/s10488-013-0528-y PMC4012002 24193818

[11] Witzel A The problem-centered interview. Forum Qualitative Sozialforschung/Forum: Qualitative Social Research (2000) 1(1):22. 10.17169/fqs-1.1.1132

[12] Böhm A Theoretical coding: text analysis in grounded theory. In: Flick U von Kardorff E Steinke I, editors. A companion to qualitative research. London: Sage (2004). p. 270–5.

[13] Strauss AL Corbin JM Grounded theory: Grundlagen qualitativer Sozialforschung. Weinheim: Beltz Psychologie Verlags Union (1996).

[14] Mey G Mruck K Grounded-theory-methodology. In: Mey G Mruck K, editors. Handbuch qualitative Forschung in der Psychologie. 1. Aufl. VS, Wiesbaden: Verl. für Sozialwiss (2010). p. 614–27. 10.1007/978-3-531-92052-8_43

[15] Corbin J Strauss A Grounded theory research: procedures, canons, and evaluative criteria. Zeitschrift für Soziologie (1990) 19(6):418–27. 10.1515/zfsoz-1990-0602

[16] atlas.ti. Available from: URL: https://atlasti.com/.

[17] Hüther FTC Jaeger S Steinert T Behandlungsverweigerung, Patientenautonomie und Zwangsmedikation [Dissertation]. Köln: Psychiatrie Verlag; Psychiatrie Verlag GmbH (2018).

[18] O’Donoghue B Roche E, Lyne J Madigan K Feeney L Service users’ perspective of their admission: a report of study findings. Ir J Psychol Med (2017) 34:251–60. 10.1017/ipm.2016.13 30115179

[19] McGuinness D Murphy K Bainbridge E Brosnan L Keys M Felzmann H Individuals’ experiences of involuntary admissions and preserving control: qualitative study. BJPsych Open (2018) 4(6):501–9. 10.1192/bjo.2018.59 PMC6293449 30564446

[20] Murphy R McGuinness D Bainbridge E Brosnan L Felzmann H Keys M Service users’ experiences of involuntary hospital admission under the Mental Health Act 2001 in the Republic of Ireland. Psychiatr Serv (2017) 68(11):1127–35. 10.1176/appi.ps.201700008 28669292

[21] Theodoridou A Schlatter F Ajdacic V Rössler W Jäger M Therapeutic relationship in the context of perceived coercion in a psychiatric population. Psychiatry Res (2012) 200(2–3):939–44. 10.1016/j.psychres.2012.04.012 22575342

[22] Paksarian D Mojtabai R Kotov R Cullen B Nugent KL Bromet EJ Perceived trauma during hospitalization and treatment participation among individuals with psychotic disorders. Psychiatr Serv (2014) 65(2):266–9. 10.1176/appi.ps.201200556 PMC4039016 24492906

[23] Lu W Mueser KT Rosenberg SD Yanos PT Mahmoud N Posttraumatic reactions to psychosis: a qualitative analysis. Front Psychiatry (2017) 8:129. 10.3389/fpsyt.2017.00129 28769826 PMC5515869

[24] Steinert T Kallert TW Medikamentöse Zwangsbehandlung in der Psychiatrie. Psychiatr Prax (2006) 33(4):160–9. 10.1055/s-2005-867054 16680623

[25] Flammer E Steinert T Das Fallregister für Zwangsmaßnahmen nach dem baden-württembergischen Psychisch-Kranken-Hilfe-Gesetz: Konzeption und erste Auswertungen. Psychiatr Prax (2019) 46:82–9. 10.1055/a-0665-6728 30149398

[26] Noorthoorn E Lepping P Janssen W Hoogendoorn A Nijman H Widdershoven G One-year incidence and prevalence of seclusion: Dutch findings in an international perspective. Soc Psychiatry Psychiatr Epidemiol (2015) 50(12):1857–69. 10.1007/s00127-015-1094-2 26188503

[27] Steinert T Lepping P Bernhardsgrütter R Conca A Hatling T Janssen W Incidence of seclusion and restraint in psychiatric hospitals: a literature review and survey of international trends. Soc Psychiatry Psychiatr Epidemiol (2010) 5(9):889–97. 10.1007/s00127-009-0132-3 19727530

[28] Acosta FJ Hernández JL Pereira J Herrera J Rodríguez CJ Medication adherence in schizophrenia. World J Psychiatry (2012) 2(5):74–82. 10.5498/wjp.v2.i5.74 24175171 PMC3782179

[29] Gibson S Brand SL Burt S Boden ZVR Benson O Understanding treatment non-adherence in schizophrenia and bipolar disorder: a survey of what service users do and why. BMC Psychiatry (2013) 13:153. 10.1186/1471-244X-13-153 23714262 PMC3695802

[30] Penttilä M Jääskeläinen E Hirvonen N Isohanni M Miettunen J Duration of untreated psychosis as predictor of long-term outcome in schizophrenia: systematic review and meta-analysis. Br J Psychiatry (2014) 205(2):88–94. 10.1192/bjp.bp.113.127753 25252316

[31] Bloch S Green SA An ethical framework for psychiatry. Br J Psychiatry (2006) 188:7–12. 10.1192/bjp.188.1.7 16388063

[32] Lysaker PH Pattison ML Leonhardt BL Phelps S Vohs JL Insight in schizophrenia spectrum disorders: relationship with behavior, mood and perceived quality of life, underlying causes and emerging treatments. World Psychiatry (2018) 17(1):12–23. 10.1002/wps.20508 29352540 PMC5775127

[33] Shiraishi N Reilly J Positive and negative impacts of schizophrenia on family caregivers: a systematic review and qualitative meta-summary. Soc Psychiatry Psychiatr Epidemiol (2019) 54(3):277–90. 10.1007/s00127-018-1617-8 30349961

[34] Awad AG Voruganti LNP The burden of schizophrenia on caregivers: a review. Pharmacoeconomics (2008) 26(2):149–62. 10.2165/00019053-200826020-00005 18198934

[35] Ranieri V Madigan K Roche E Bainbridge E McGuinness D Tierney K Caregivers’ perceptions of coercion in psychiatric hospital admission. Psychiatry Res (2015) 228(3):380–5. 10.1016/j.psychres.2015.05.079 26163727

[36] Reisch T Beeri S Klein G Meier P Pfeifer P Buehler E Comparing attitudes to containment measures of patients, health care professionals and next of kin. Front Psychiatry (2018) 9:529. 10.3389/fpsyt.2018.00529 30416459 PMC6212593

[37] Jankovic J Yeeles K Katsakou C Amos T Morriss R Rose D Family caregivers’ experiences of involuntary psychiatric hospital admissions of their relatives—a qualitative study. PLoS ONE (2011) 6(10):e25425. 10.1371/journal.pone.0025425 22022393 PMC3192057

[38] Steinert T Hirsch S, editors. S3-Leitlinie Verhinderung von Zwang: Prävention und Therapie aggressiven Verhaltens bei Erwachsenen. 1. Auflage 2019. Berlin: Springer (2019). 10.1007/s00115-019-00801-2 31473766

[39] Krieger E Moritz S Weil R Nagel M Patients’ attitudes towards and acceptance of coercion in psychiatry. Psychiatr Res (2018) 260:478–85. 10.1016/j.psychres.2017.12.029 29287276

[40] Price O Baker J Bee P Grundy A Scott A Butler D Patient perspectives on barriers and enablers to the use and effectiveness of de-escalation techniques for the management of violence and aggression in mental health settings. J Adv Nurs (2018) 74(3):614–25. 10.1111/jan.13488 29082552

[41] Tingleff EB Bradley SK Gildberg FA Munksgaard G Hounsgaard L “Treat me with respect”. J Psychiatr Ment Health Nurs (2017) 24(9–10):681–98. 10.1111/jpm.12410 28665512

[42] Priebe S Dimic S Wildgrube C Jankovic J Cushing A McCabe R Good communication in psychiatry—a conceptual review. Eur Psychiatry (2011) 26(7):403–7. 10.1016/j.eurpsy.2010.07.010 21571504

[43] Carpenter WT Gold JM Lahti AC Queern CA Conley RR Bartko JJ Decisional capacity for informed consent in schizophrenia research. Arch Gen Psychiatry (2000) 57(6):533–8. 10.1001/archpsyc.57.6.533 10839330

